# *Dendrobium huoshanense* C.Z.Tang et S.J.Cheng: A Review of Its Traditional Uses, Phytochemistry, and Pharmacology

**DOI:** 10.3389/fphar.2022.920823

**Published:** 2022-07-12

**Authors:** Leilei Gao, Fang Wang, Tingting Hou, Chunye Geng, Tao Xu, Bangxing Han, Dong Liu

**Affiliations:** ^1^ College of Biological and Pharmaceutical Engineering, West Anhui University, Lu’an, China; ^2^ Anhui Province Traditional Chinese Medicine Resource Protection and Sustainable Utilization Engineering Laboratory, Lu’an, China; ^3^ School of Pharmacy, Anhui University of Chinese Medicine, Hefei, China

**Keywords:** *Dendrobium huoshanense*, phytochemistry, pharmacology, materia medica research, resource distribution

## Abstract

*Dendrobium huoshanense*, a traditional medicinal and food homologous plant, belongs to the family Orchidaceae and has a long history of medicinal use. It is reported that the stem of *D. huoshanense* has a variety of bioactive ingredients such as polysaccharides, flavonoids, sesquiterpenes, phenols, etc. These bioactive ingredients make *D. huoshanense* remarkable for its pharmacological effects on anti-tumor, immunomodulation, hepatoprotective, antioxidant, and anticataract activities. In recent years, its rich pharmacological activities have attracted extensive attention. However, there is no systematic review focusing on the chemical compositions and pharmacological effects of *D. huoshanense*. Therefore, the present review aims to summarize current research on the chemical compositions and pharmacological activities of *D. huoshanense*. This study provides valuable references and promising ideas for further investigations of *D. huoshanense*.

## Introduction

**FIGURE 1:**
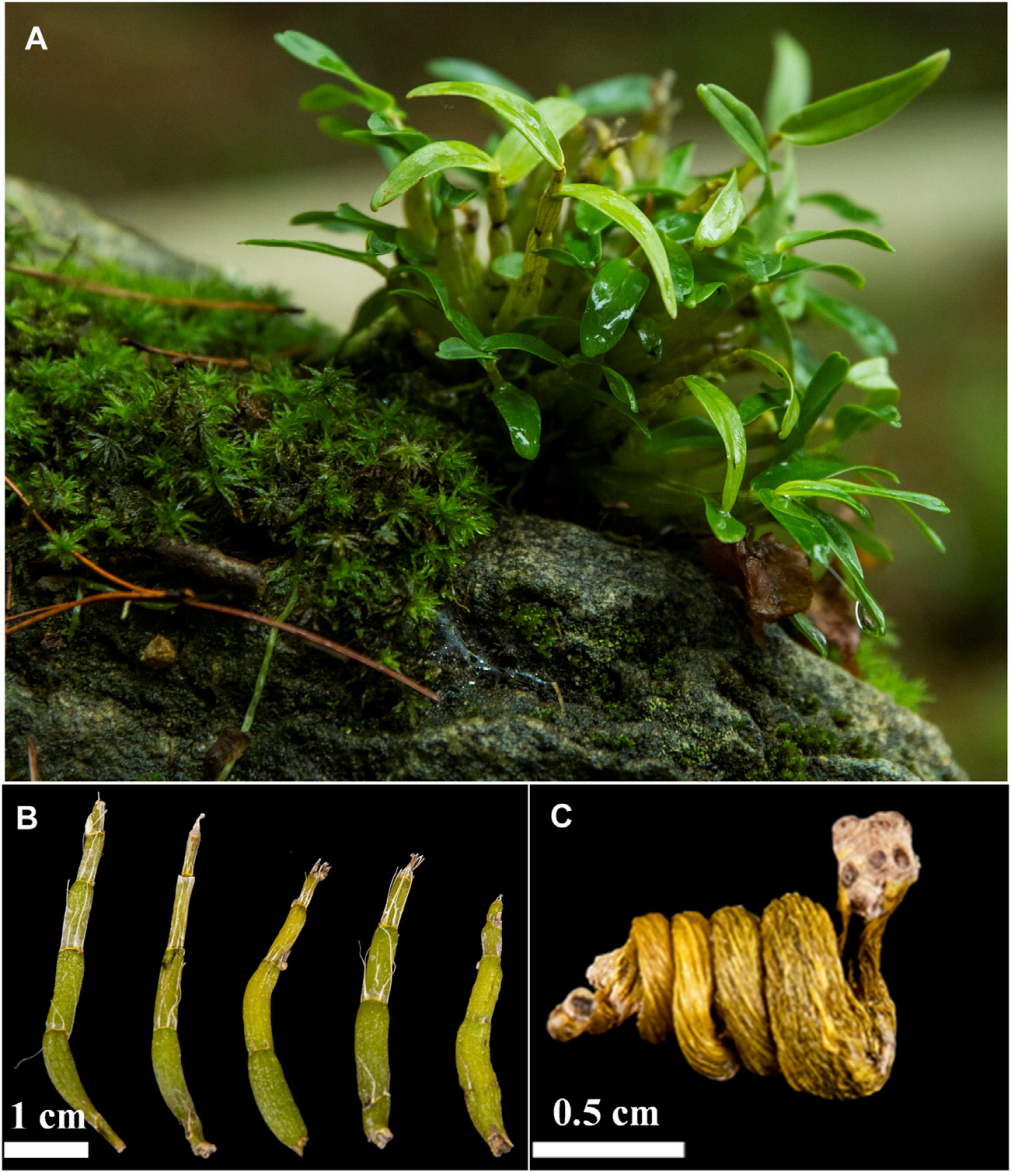
The whole plant **(A)**, fresh strips **(B)**, and the commercial product named *Fengdou* **(C)** of *D. huoshanense*.

*Dendrobium* is a valuable traditional Chinese medicine with a long history of medicinal use (Li et al., 2022; Zhu et al., 2021; Niu et al., 2018). It was first recorded in “Shennong’s Herbal Classic” (Chen et al., 2021). However, there are various species in this genus, and their quality varies significantly (Chen et al., 2011). *Dendrobium huoshanense* C.Z.Tang et S.J.Cheng is an endemic epiphytic orchid species as well as a national geographical indication product of China (Figure 1) (Huang et al., 2007). This plant was first described in a local classic reference “Bai Cao Jing” (《百草镜》) and the distribution area of this plant is in the Da-bie Mountains and adjacent areas, especially in the Huoshan County town, Anhui province, China (Zhao et al., 2017; Yuan et al., 2019; Chen et al., 2020). Currently, the species has been included in the Pharmacopoeia of the Peoples Republic of China (2020 Edition) and approved to be used as food (Hao et al., 2018; Editorial Board of China Pharmacopoeia Committee 2020; Hao et al., 2021c).

*D. huoshanense* is locally known as “Mihu,” and its stem has high medicinal value, which is sweet in taste and slightly cold in nature. It is commonly utilized for benefiting the stomach and producing body fluid, clearing heat, and nourishing yin (Hsieh et al., 2008; Wang et al., 2009; Dai et al., 2021). Previous studies have showed that *D. huoshanense* has various activities, such as immunoregulation, anti-oxidation, anti-cataract, anti-glycation, anti-aging, anti-cataract, antitumor, anti-rheumatoid arthritis, anti-atherosclerosis, anti-inflammation, hypoglycemic activity, and liver protection activities (Wu et al., 2009; Ohara et al., 2013; Deng et al., 2016; Zhang et al., 2020; Hao J.-W. et al., 2021; Zhu et al., 2022). The main components that play a therapeutic role are active substances such as flavonoids, sesquiterpenes, and especially polysaccharides, which is the index of the quality evaluation of *D. huoshanense* (Lee and Chen 2014; Liu et al., 2018; Yuan et al., 2018; Chen et al., 2019; Zhou et al., 2020; Hao et al., 2021a; Wang et al., 2021). These diverse chemical compositions and extensive pharmacological activities of *D. huoshanense* have attracted much attention, while posing great difficulties for further research.

At present, there is much literature summarizing the *Dendrobium* genus, but no literature systematically reviewing the status of the research on *D. huoshanense*. Therefore, this review systematically describes the current research status of a specific species, *D. huoshanense*, including the materia medica research, resource, phytochemistry, and pharmacology, to provide a reference for further research on *D. huoshanense*.

## Materia Medica Research

### Materia Medica Research on Origin

The habitats of *D. huoshanense* have been recorded in many important classical documents of traditional Chinese medicine. In the period of 220–450 AD, “Records of Famous Doctors” (《名医别录》) recorded that “*Dendrobium* grows on the stone beside the water in the valley of Lu’an”. It is found that Lu’an refers to the area of Huoshan in the Anhui province at that time, demonstrating that the earliest recorded origin of *D. huoshanense* is in the Huoshan County. “Bai Cao Jing” (《百草镜》) in the Qing Dynasty described that *Dendrobium* from the Lu’an and Huoshan Counties was called “Huoshan Shihu” and was the best, which was first documented with the name of *D. huoshanense*. In 1984, Tang et al. systematically studied several species of *Dendrobium* and analyzed their botanical traits (Tang and Cheng 1984). Moreover, “Mihu” produced in the Huoshan County was officially named *Dendrobium huoshanense* C.Z. Tang et S.J. Cheng.

At present, “Flora Reipublicae Popularis Sinicae” reported that “*D. huoshanense* is produced in southwestern Henan (Nanzhao) and southwestern Anhui (Huoshan) of China. It grows on tree trunks in mountain forests and on rocks in valleys. The type specimen was collected in Anhui (Huoshan) of China”. Through materia medica research and field investigations, Liu and wang et al. also clearly support the occurrence of *D. huoshanense* in Huoshan, Anhui province of China (Liu 1996; Wang and Peng 2004). In 2007, *D. huoshanense* was listed in the national geographic indication to protect products. In short, the habitats of *D. huoshanense* are mainly concentrated in Huoshan, Anhui province of China.

### Materia Medica Research on Medicinal Use

*D. huoshanense* has been used in medicine for a long time. “Records of Famous Doctors” (《名医别录》) recorded that *Dendrobium* could nourish the essence, reinforce the kidneys, calm the stomach, build muscles, relieve foot and knee pain, and remove convulsions, while emphasizing that the origin of *Dendrobium* is the now Huoshan County. These are enough to show that the orchid plant recorded in “Records of Famous Doctors” (《名医别录》) refers to *D. huoshanense* (Tao 2013). The ancient medical reference “Bai Cao Jing” (《百草镜》) put forward the local name of *D. huoshanense* for the first time and stated that this plant is the best for medicinal use. The traits of *D. huoshanense*, including dwarf plantlets in a cluster and an upright or bending stem resembling grasshopper legs, are almost identical to those recorded in the “Shen Nong Ben Cao” (Zhao 1998). It can be seen that *D. huoshanense* has been used as high-quality *Dendrobium* for medicinal purposes for many generations. In addition, *D. huoshanense* is included in the Chinese Pharmacopoeia (2020 version), and its efficacy is recorded for benefiting the stomach and producing body fluids, clearing heat, and nourishing the yin (Editorial Board of China Pharmacopoeia Committee 2020).

## Resource

### Resource Distribution

*Dendrobium* is the second largest genus belonging to the family Orchidaceae, widely distributed in Asia, Europe, Oceania, and other regions (Liu JL. et al., 2020; Han et al., 2020; Hu et al., 2020; Wang et al., 2020). There are 105 species and two varieties of *Dendrobium* in China, which are produced in Anhui, Zhejiang, Jiangxi, Fujian, Taiwan, Hubei, Hunan, Guangdong, Guangxi, Hainan, Sichuan (including Chongqing), Guizhou, Yunnan, Tibet, etc. Among them, there are 39 species of *Dendrobium* with medicinal purposes, including *D. huoshanense* (Chiang et al., 2012; Ye et al., 2021). However, the natural distribution of *D. huoshanense* is relatively narrow, because of its preference for a cool and moist environment, and high environmental requirements (Liu et al., 2007; Qin et al., 2008; Niu et al., 2020). Wild *D. huoshanense* mainly grows as an epiphytic on the cliffs beside rivers and valleys at a slight altitude ranging between 200 and 1,200 m. In view of its strict requirements on the environment, *D. huoshanense* is currently endemic to the Da-bie Mountains, with Huoshan in the Anhui Province as the center of distribution (Zha et al., 2007d; Zheng et al., 2011; Wang et al., 2012; Wu et al., 2016). The cultivation industry of *D. huoshanense* is also in Huoshan, which has reached 8.0 million m^2^ with 350 tons of the annual production (including flower, fresh and dry materials of *D. huoshanense*).

### Resource Conservation

Over the years, due to the unreasonable collection of *D. huoshanense* and the limitation of its own reproduction modes, the wild resources are on the verge of extinction. It has been listed in the National Key Protected Wild Plants as a “Class I protected species” (http://www.gov.cn/zhengce/zhengceku/2021-09/09/content_5636409.htm). In order to improve resource conservation and resolve the market demand, key measures must be taken. By mastering the distribution, reserves, and the native environment of *D. huoshanense*, the provenance protection base has been established and some local standards such as “*Dendrobium huoshanense* C. Z. Tang et S. J. Cheng” and “Technical regulation for the protection of the protospecies of *Dendrobium huoshanense* C. Z. Tang et S. J. Cheng” have been promulgated. In addition, the rapid breeding of *D. huoshanense* has been realized through modern biotechnology, which effectively alleviates the resource situation of *D. huoshanense* (Luo et al., 2003; Shi et al., 2003).

**FIGURE 2:**
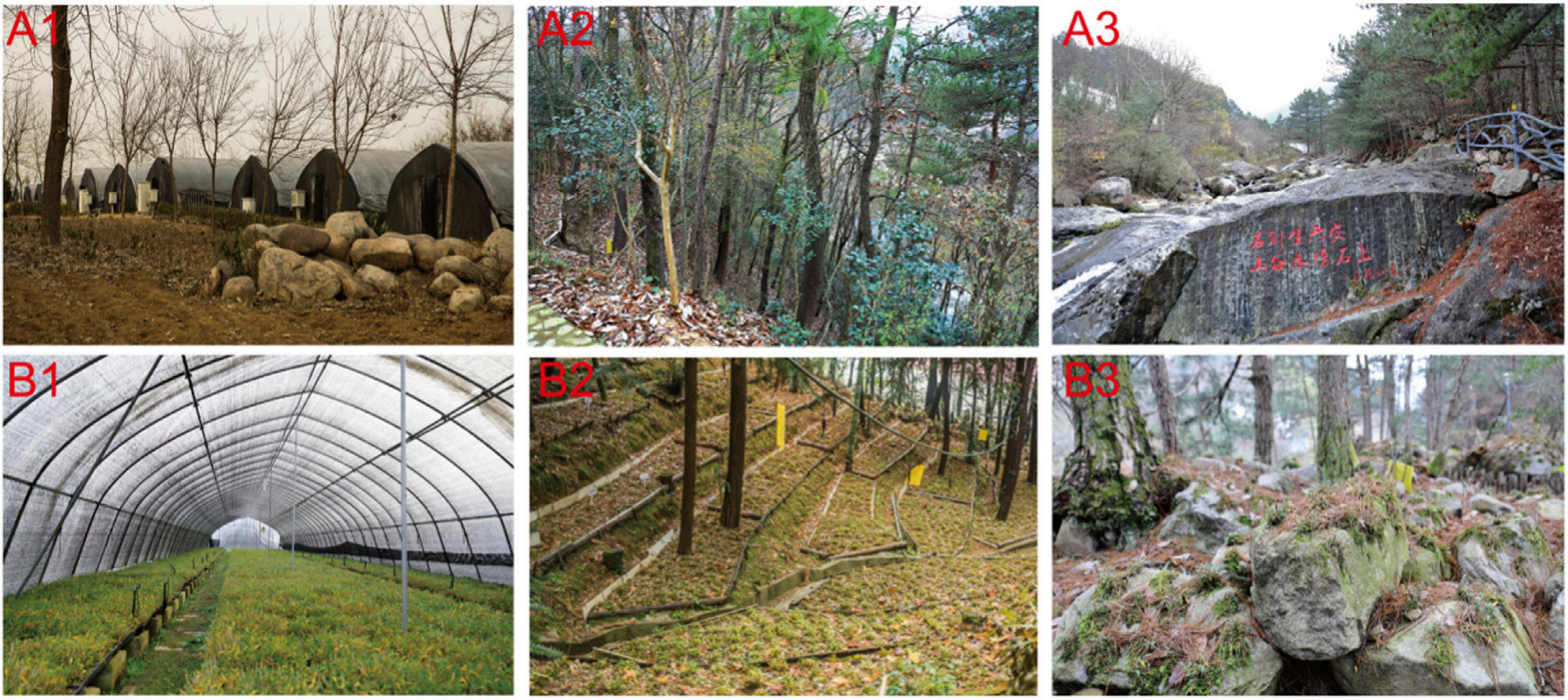
External **(A1–A3)** and internal **(B1**–**B3)** growth environments of *D. huoshanense* under the facility cultivation mode, under-forest cultivation mode, and simulative habitat cultivation mode, respectively.

Scientific-based harvesting is also an important factor for resource protection and reserve. The suitable harvesting time for *D. huoshanense* is from November to June of the following year, and there are two ways for harvesting: “Cai Lao Liu Xin” and whole plant harvesting (Ren et al., 2014). “Cai Lao Liu Xin” is the method of harvesting certain stems with ages of more than 3 years old, while whole plants should be harvested from clumps of more than 20 months old. Furthermore, the cultivation modes of *D. huoshanense* have been systematically researched, including facility cultivation mode, under-forest cultivation mode, and simulative habitat cultivation mode, and provide powerful help for the conservation resources of *D. huoshanense* (Figure 2) (Yi et al., 2021). All in all, the resources of *D. huoshanense* have been effectively protected and rationally exploited.

## Constituents From *D. huoshanense*

Modern biomedical researches show that *D. huoshanense* contains many active ingredients, such as polysaccharides, flavonoids, bibenzyls, phenols, sesquiterpenoids, etc. It is confirmed that these active ingredients are used singly or in combinations to make *D. huoshanense* of high medicinal value. The following sections will elaborate on the bioactivity present in between the ingredients and *D. huoshanense*.

### Polysaccharides

Polysaccharide is the main active ingredient of *D. huoshanense*, which is effective in anti-inflammation, anti-oxidant, anti-tumor, and immunoregulation, and is mainly composed of glucose, galactose, xylose, and arabinose and mannose, with the molecular weight distribution range of 3,200–8,090,000 Da (Zha et al., 2007b; Xie et al., 2018; Hao et al., 2019; Dai et al., 2020; Yi et al., 2021b; Zhang Y. et al., 2021; Wu et al., 2022). At present, a variety of *D. huoshanense* polysaccharides have been isolated, such as DHAH (Meng et al., 2013), DHP (Pan et al., 2014), DHP1A (Tian et al., 2013), HPS-1B23 (Zha et al., 2007c), DHP-W2 (Pan et al., 2013), DHPD1 (Zha et al., 2013), DHPD2 (Li et al., 2014), TC-DHPA4 (Si et al., 2018), GXG (Xie et al., 2019a), cDHPS (Liu B. et al., 2020; Shang et al., 2021), and DHP-4A (Li et al., 2015). Their structures are identified by GC-MS, HPLC-GPC, IR, NMR, HSQC, and HMBC, and the details are shown in Table 1.

**TABLE 1:** Summary of polysaccharides from D. huoshanense.

Compound name	Monosaccharide composition	Structural features	Molecular weight (Da)	References
DHAH	Man: Glc: Gal = 100:53:02	ND	2.2×10^5^	Meng et al. (2013)
DHP	Ara: Gal: Glc: Man = 0.03:0.11:1.00:0.07	ND	ND	Pan et al. (2014)
DHP1A	Man: Glc: Gal = 2.5:16:1	(1→4)-linked α-D-Glc*p*, (1→6)-linked α-D-Glc*p* and (1→4)-linked β-D-Man*p*	6.7×10^3^	Tian et al. (2013)
HPS-1B23	Glc: Man: Gal = 31:10:8	(1→4)-linked α-D-Glc*p*, (1→6)-linked α-D-Glc*p*, (1→3,6)-linked α-D-Glc*p* and (1→3,6)-linked α-D-Man*p*	2.2×10^4^	Zha et al. (2007c)
DHP-W2	Glc: Xyl: Gal = 1.0:1.0:0.5	(1→6)-linked β-D-Glc*p*, (1→4)-linked β-D-Glc*p* and (1→4,6)-linked β-D-Glc*p* with branches at O-4/6	7.3×10^4^	Pan et al. (2013)
DHPD1	Glc: Ara: Gal = 0.023:1.023:0.021	ND	3.2×10^3^	Zha et al. (2013)
DHPD2	Gala: Glc: Ara = 0.896:0.723:0.2	(1→5)-linked α-L-Ara*f*, (1→6)-linked α-D-Glc*p*, (1→6)-linked β-D-Glc*p*, (1→4)-linked β-D-Glc*p*, (1→3,6)-linked β-D-Gal*p* and (1→6)-linked β-D-Gal*p*, with the branches of terminal α-D-Xly*p* and β-D-Man*p*.	8.09×10^6^	Li et al. (2014)
TC-DHPA4	Rha: Ara: Man: Glc: Gala = 1.28:1:1.67:4.71:10.43	(1→6)-linked β-Gal*p*, (1→4)-linked β-Glc*p*, and (1→6)-linked β-Glc*p*, with four branched chains	8.0 × 10^5^	Si et al. (2018)
GXG	Glc: Xyl: Gal = 2.85 : 2.13 : 1.00	(1→4)-linked Xyl*p*, (1→2,4)-linked Xyl*p*, (1→4)-linked Gal*p*, (1→3,6)-linked Gal*p*, (1→6)-linked Glc*p*, (1→4)-linked Glc*p* and (1→4,6)-linked Glc*p*	1.78×10^6^	Xie et al. (2019a)
Cdhps	Man: Glc = 2.88:1.00	β-(1→4)-linked D-Glc*p* and β-(1→4)-linked D-Man*p* with partial acetylation at 3-OH	2.59×10^5^	(Liu et al., 2020a; Shang et al., 2021)
DHP-4A	Glc:Ara: Man: Rha = 13.8:3.0:6.1:2.1	(1→6)-linkedβ-D-Glc*p*, (1→6)-linkedβ-D-Man*p* and (1→3,6)-linked β-D-Man*p*, with branches at the C-3 position of (1→6)-linkedβ-D-Man*p*	2.32×10^5^	Li et al. (2015)

Ara, arabinose; Man, mannose; Glc, glucose; Gal, galactose; Xyl, xylose; Rha, rhamnose.

### Sesquiterpenoids

**FIGURE 3:**
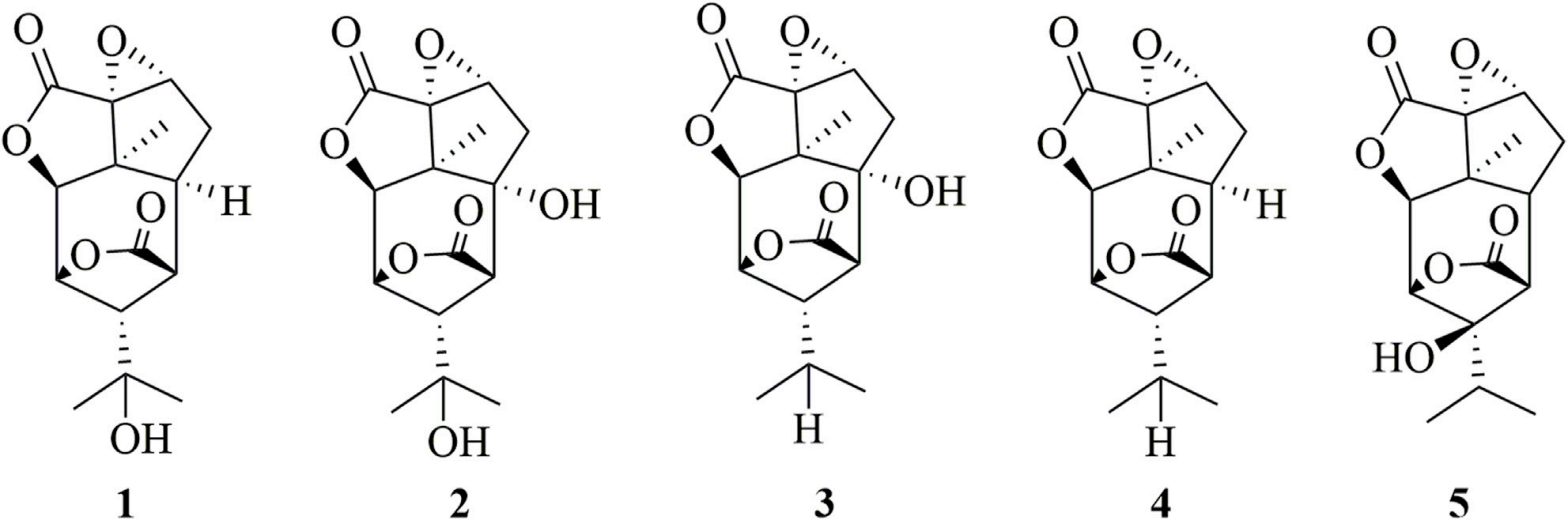
Structure of sesquiterpenes (**1–5**) in *D. huoshanense*.

Sesquiterpenoids are essential chemical components, which are also the significant material basis for the pharmacological activity of *Dendrobium* (Fan et al., 2021). Sesquiterpenoids (**1–5**) have also been found in *D. huoshanense* and their structures have been elucidated through extensive spectroscopic analyses. The detailed structural information is shown in Figure 3 (Chen et al., 2022).

### Flavonoids

**FIGURE 4:**
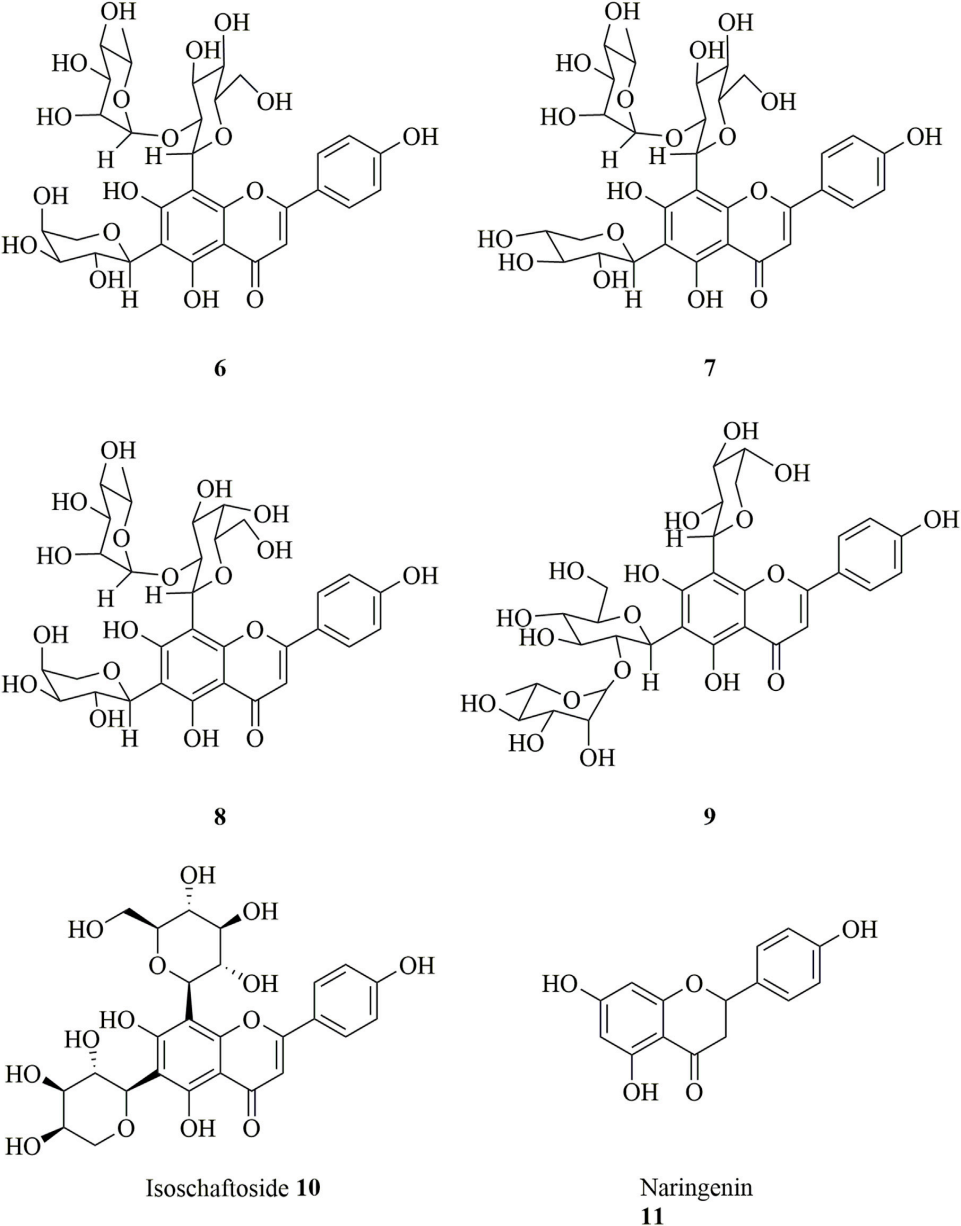
Structure of flavonoids (**6–11**) in *D. huoshanense*.

Flavonoids, which are the most widely distributed class of compounds in Chinese herbal medicine, are important chemical constituents of *Dendrobium* (Yuan et al., 2022). Flavonoids are also found in *D. huoshanense*, but only few flavonoids and flavonoid glycosides (**6–11**) have been isolated, and their structures are shown in Figure 4 (Chang et al., 2010; Zhao et al., 2021).

### Bibenzyls

**FIGURE 5:**
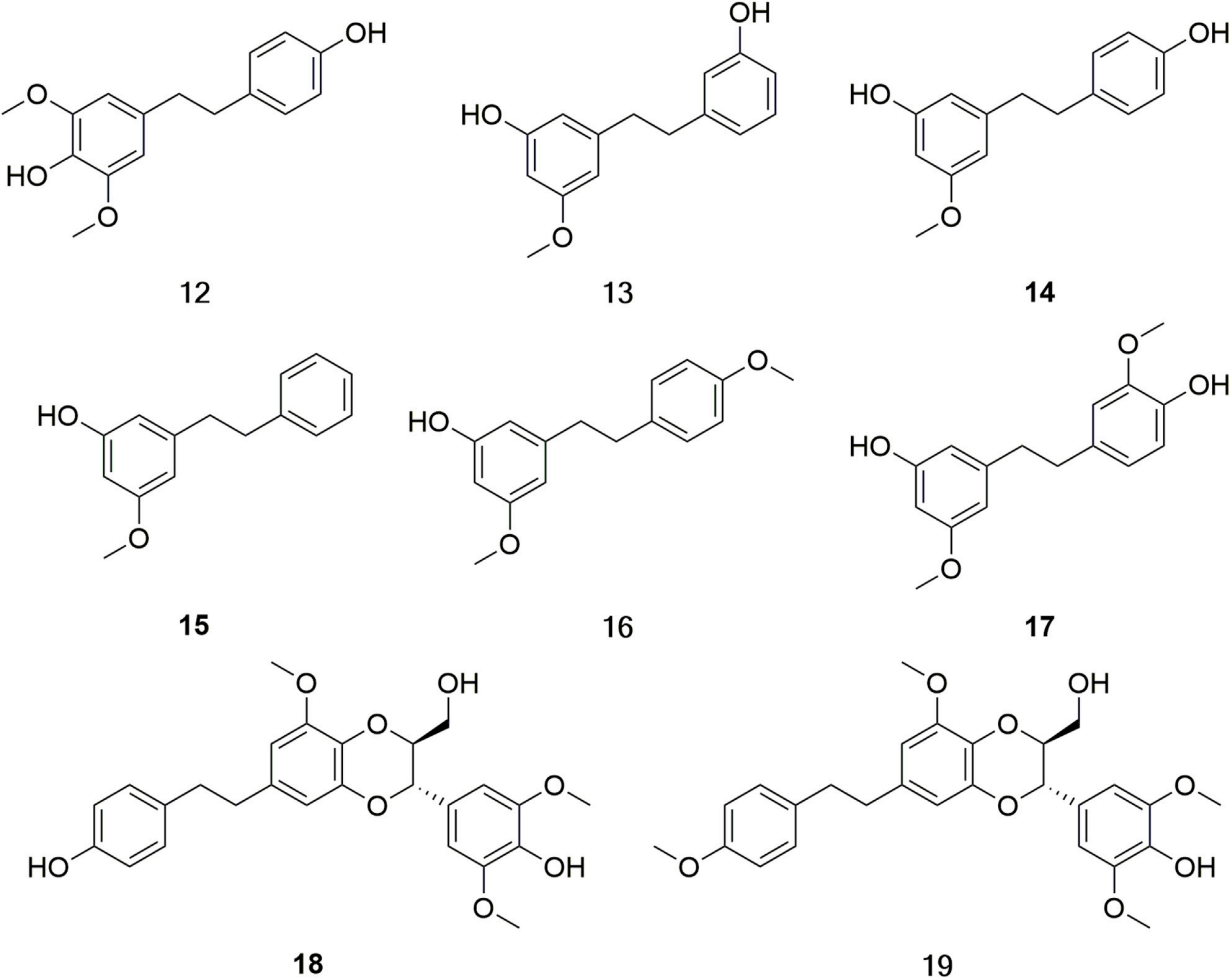
Structure of bibenzyls (**12–19**) in *D. huoshanense*.

Bibenzyls, with a basic structural skeleton 1, 2-diphenylethane, are widely distributed in *Dendrobium* (Sun et al., 2021). Currently, few bibenzylates have been isolated from *D. huoshanense*. According to the literature statistics, eight bibenzylates (**12–19**) have been isolated and identified from *D. huoshanense* (Figure 5) (Li QM. et al., 2020; Zhao et al., 2021).

### Phenols

**FIGURE 6:**
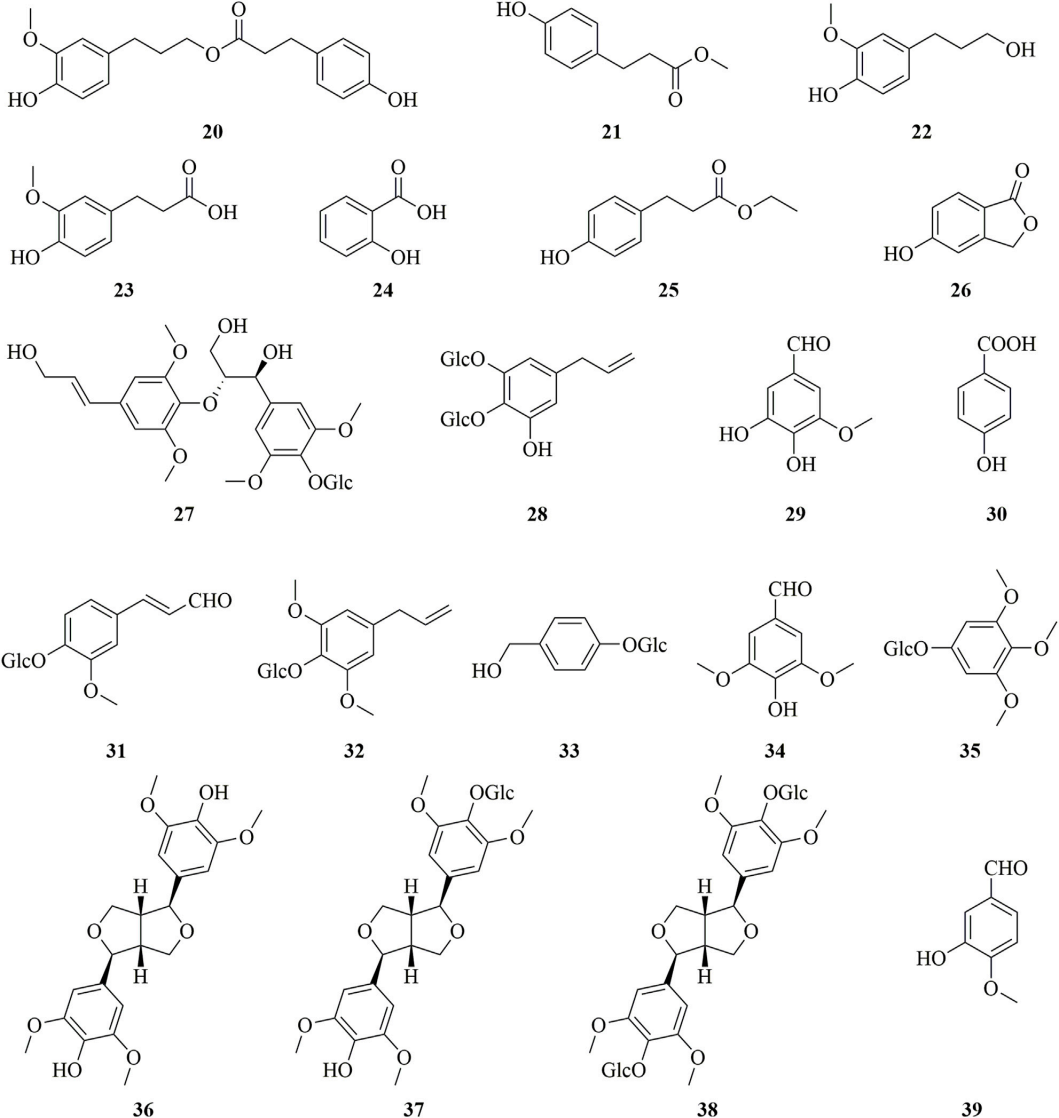
Structure of phenols (**20–39**) in *D. huoshanense*.

Phenols are widely present in *Dendrobium* and have complex and diverse structures, but are not the main active components (Dong et al., 2020). *D. huoshanense* also contains a large number of phenols, and 20 phenols have been isolated. Their chemical structures are shown in Figure 6 (Chang et al., 2010; Zhao et al., 2021).

### Other Compounds

**FIGURE 7:**
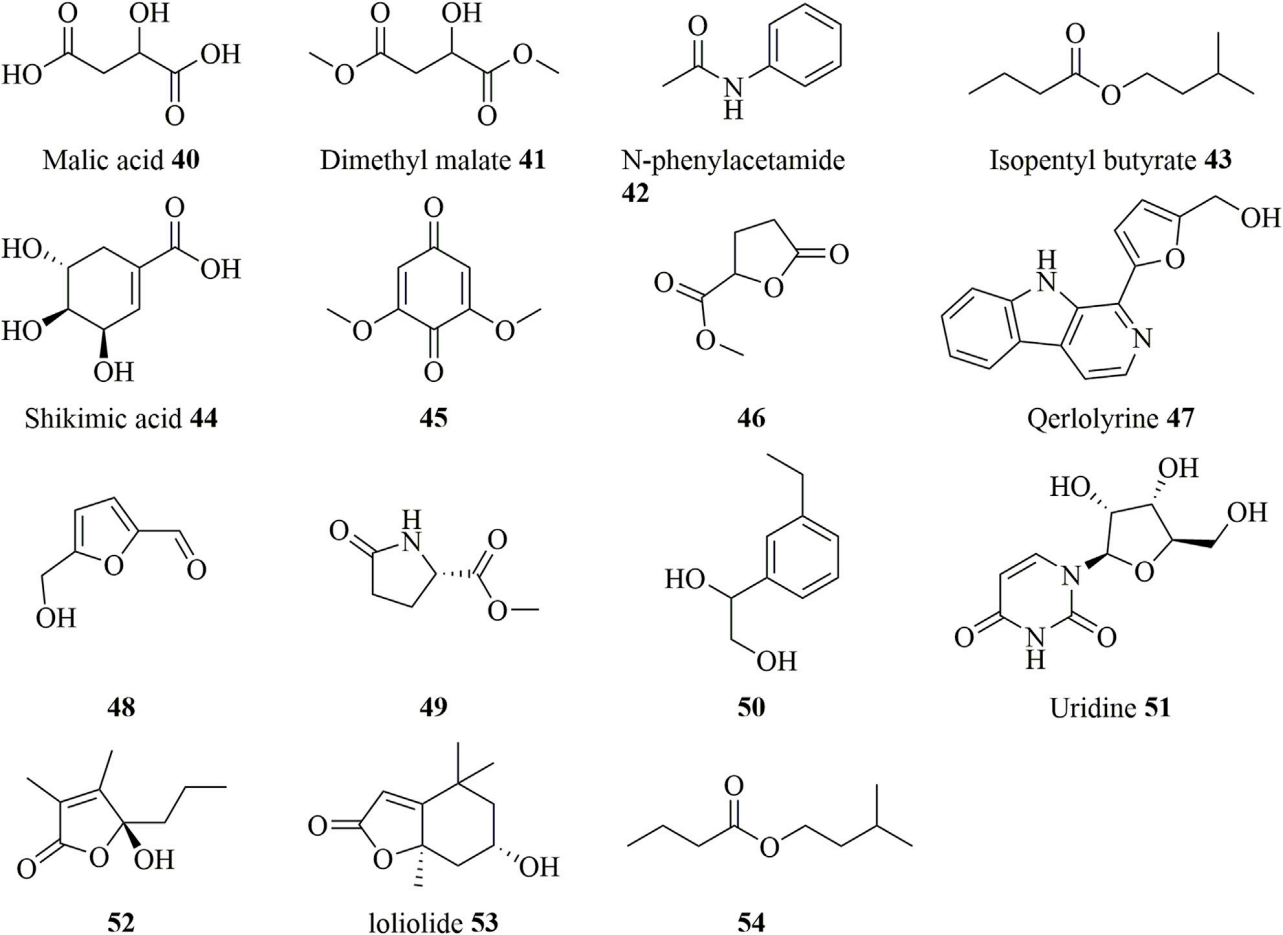
Structure of other compounds (**40–54**) in *D. huoshanense*.

In addition to the aforementioned chemical constituents, *D. huoshanense* also contains other types of chemical constituents (Chang et al., 2010; Zhao et al., 2021), including Malic acid, Dimethyl malate, N-phenylacetamide, Isopentyl butyrate, Shikimic acid, etc., and their chemical structures are shown in Figure 7.

## Pharmacology

The active components in *D. huoshanense* are diverse and complex, and have many important effects. The pharmacological effects and possible mechanisms of action of *D. huoshanense* will be elaborated and reviewed below.

### Antitumor Activity

As a special species of the *Dendrobium* genus, *D. huoshanense* has the ability to improve body function to prevent and treat tumor diseases. In the cell proliferation assay, the aqueous extract of *D. huoshanense* could effectively inhibit the growth of HeLa cells with a concentration range of 2–10 mg/ml (Zhang X. et al., 2021). Moreover, the inhibition rates of the aqueous extract of different plant ages on HeLa cells were varied. Among them, the aqueous extract of a 3 year old *D. huoshanense* possessed the highest anticancer activity, followed by a 2 year old, and the annuals were the least active.

In addition to the crude extract of *D. huoshanense*, polysaccharides and small molecule compounds isolated from *D. huoshanense* also have good antitumor activities. Luo et al. investigated the inhibitory effect of *D. huoshanense* polysaccharide cDHPS on gastric cancer and preliminarily explored its constitutive relationship. The results showed that cDHPS (at 0.2 mg/ml) could significantly inhibit tumor growth, induce tumor cell apoptosis, suppress tumor angiogenesis, and enhance a T cell immune response of murine forestomach carcinoma tumor-bearing mice (Liu B. et al., 2020; Liu et al., 2021). The structure–activity relationship investigation indicated that the molecular weight and the O-acetyl group of *D. huoshanense* polysaccharides greatly influenced the anti-gastric cancer activity.

Xu et al. isolated five picrotoxane-type sesquiterpenes (**1–5**) from *D. huoshanense* and examined their cytotoxicity activity on HL-60, MCF-7, SMMC-7721, and SW-480 human cancer cells (Chen et al., 2022). The results showed that compound **3** (Figure 3) showed significant effects on HL-60, MCF-7, SMMC-7721, and SW-480 cells with IC50s of 5.81, 6.49, 9.65, and 6.80 μM, respectively. Importantly, the cytotoxicity activity of compound **3** was comparable to 5-fluorouracil. To sum up, sesquiterpenes from *D. huoshanense* were worth further studying to find novel anticancer drugs.

### Antioxidant Activity

Many traditional Chinese medicines have been reported to possess antioxidant activity (Muhammad et al., 2022). Compared with synthetic antioxidants, herbal components have the advantage of less toxicity, so they have attracted much attention. *D. huoshanense*, an important traditional Chinese medicine, has also shown a significant antioxidant activity. Luo et al. compared the antioxidant activity of crude polysaccharides of *D. huoshanense* with those crude polysaccharides of *D. officinale*, *D. nobile,* and *D. chrysotoxum* (Pan et al., 2014). The results showed that *D. huoshanense* polysaccharide had the strongest antioxidant activity and could significantly enhance the activities of antioxidative enzymes superoxidedismutase (SOD) and catalase (CAT) and increase the content of L-glutathione (GSH). In addition, refined *D. huoshanense* polysaccharides were isolated to investigate the antioxidant activity (Xu et al., 2019). It was found that a refined *D. huoshanense* polysaccharide could reduce malonaldehyde (MDA) levels, increase T-AOC levels, alleviate D-galactose-induced oxidative damage in mice, and exhibit a significant antioxidant activity.

Luo et al. have also proved that DHP1A obtained from *D. huoshanense* possesses a remarkable inhibition effect on the lipid peroxidation *in vitro* (Tian et al., 2013). At a concentration of 2.0 mg/ml, the inhibition rate of DHP1A reached 56.5%, which was higher than that of dextran (*p* < 0.01) and close to that of vitamin C. Furthermore, DHP1A could alleviate the hepatic oxidative stress caused by CCl_4_ in mice. These findings demonstrated that DHP1A exhibited a significant antioxidant activity and its mechanism of action might be anti-lipid peroxidation.

### Immunomodulatory Activity

Immune function is the body’s resistance to diseases, improving the immunity of the body and enhancing the immune function is the key to prevent the occurrence of diseases and restore health. *D. huoshanense* has an excellent performance in immune regulation. For example, Wong et al. demonstrated that the administration of a crude polysaccharide of *D. huoshanense* (DH-PS) in mice not only induced the production of cytokines (IL-12 p40, IL-6, IL-10, and TNF-α) and chemokines (KC, RANTES, MCP-1, and MIP-1β), but also activated or amplified various immune cells, including NK cells/activated NK cells, NKT cells/activated NKT cells, regulatory T cells, B cells/activated B cells, CD4^+^ T cells/activated CD4^+^ T cells, and CD8^+^ T cells/activated CD8^+^ T cells (Lin et al., 2014). These results indicate that DH-PS regulated the immune function through stimulating cytokine secretions and promoting the expansions and/or activations of immune cells.

Luo et al. have investigated the immune activity of polysaccharides from *D. huoshanense* at different growth stages (Chen et al., 2012). The results showed that the polysaccharides obtained from different growth stages of *D. huoshanense* could promote the production of interferon-γ (IFN-γ) and tumor necrosis factor-α (TNF-α) in mouse spleen cells, showing similar immune activities. Later, some authors confirmed that the water-soluble polysaccharides HPS-1B23, HPS1A, HPS1B, and HPS2B isolated from *D. huoshanense* exhibited immunomodulatory activities, through stimulating macrophages to secrete IFN-γ and TNF-α (Zha, Luo and Jiang 2007a; Zha et al., 2007c). Further study revealed that HPS2B23 activated macrophages by binding to toll-like receptor 4 and triggering nuclear factor-kappa B (NF-κB), mitogen-activated protein kinases (MAPKs), and phosphoinositide 3-kinase-Akt (PI3 K/Akt) signaling pathways (Xie et al., 2016). In addition, HPS-1B23 has also been shown to modulate the small intestinal immune system and the systemic immune system after oral administration (Zha et al., 2014). Luo et al. also fractionated the homogeneous polysaccharides DHP-4A and GXG from *D. huoshanense*, which showed significant immunomodulatory effects (Li et al., 2015; Xie et al., 2019b). In general, *D. huoshanense* polysaccharides have a significant activity in immunomodulation but the structure–activity relationship has not been systematically studied.

### Protective Effects on Liver

The liver, which is the largest digestive and metabolic organ in the body, can metabolize endogenous or exogenous toxic substances and is highly susceptible to damage by various toxic substances. Therefore, it is especially necessary to improve the protection of the liver. In previous studies, our research team investigated the protective effect of *D. huoshanense* on liver injuries in mice. First, we compared the effect of *D. huoshanense* with the other four species of *Dendrobium* (*D. officinale* (Huoshan), *D. officinale* (Yunnan), *D. moniliforme,* and *D. henanense*) (Wang et al., 2017). It was found that the administration of freshly squeezed juices of *Dendrobium* at the dose of 1.25 and 7.5 g‧kg^−1^ for 2 weeks had a protective effect on CCl_4_-induced acute liver injury, but the effect was different, among which, *D. huoshanense* had the significant activity compared with others. Thereafter, the hepatoprotective effects of *D. huoshanense* with different cultivation patterns and growth years were also investigated. The results showed that *D. huoshanense* cultivated in under-forest cultivation planting had better protective effects on acute liver injuries induced by carbon tetrachloride, acetaminophen, and cyclophosphamide in mice than *D. huoshanense* cultivated in greenhouse (Li Z. Q. et al., 2020). In addition, *D. huoshanense* plants of different ages could reduce the acute liver injury induced by acetaminophen in mice, where the 2-year old *D. huoshanense* had the best efficacy (Li et al., 2021).

Luo et al. investigated that the protective effects of crude polysaccharides from *D. huoshanense* on CCl_4_-induced acute liver injuries in mice (Huang et al., 2013). The results showed that the crude polysaccharides with different doses (200, 100, and 50 mg‧kg^−1^) could reduce the activity of alanine aminotransferase (ALT) and aspartate aminotransferase (AST) in the serum and the level of hepatic MDA, enhance the activity of hepatic SOD, inhibit the expression of TNF-α in hepatocytes, and alleviate liver tissue damage induced by CCl_4_, indicating that the *D. huoshanense* polysaccharide could protect mice from CCl_4_-induced acute liver injury by scavenging free radicals and inhibiting lipid peroxidation and the expression of TNF-α. Furthermore, Luo et al. further found that refined polysaccharides, galactoglucomannan form *D. huoshanense*, dose-dependently inhibited the activity of ALT, AST, and lactate dehydrogenase (LDH). At a dose of 200 mg‧kg^−1^‧day^−1^, galactoglucomannan significantly decreased the selenite-increased activities of ALT, AST, and LDH, and reduced the MDA levels and H_2_O_2_ to 59.5% and 34.6%, respectively (Pan et al., 2012). In addition, galactoglucomannan could reverse the selenium-induced decrease of the concentration of GSH in the liver and inhibit selenium-induced transforming growth factor β1 (TGF-β1) expression, showing significant potential to prevent liver injury and fibrosis. Interestingly, the daily supplementation of galactoglucomannan prevents ethanol-induced liver injury. The results of the proteomic analysis and metabolomic analysis indicated that galactoglucomannan might correct the perturbed metabolism pathways by ethanol exposure to protect the liver from ethanol-induced injuries (Wang et al., 2014; Wang et al., 2015).

DHP1A, a polysaccharide isolated from *D. huoshanense*, could also reduce the levels of ALT, AST, LDH, and 8-hydroxy-20-deoxyguanosine (8-OhdG) in the serum, exhibiting a strong hepatoprotective activity. Meanwhile, DHP1A also down-regulated the expression of TNF-α, interleukin-1β (IL-1β), monocyte chemoattractant protein-1 (MCP-1), macrophage inflammatory protein-2 (MIP-2), CD68, and phosphorylated IκBα (p-IκBα), demonstrating that the hepatoprotective activity of DHP1A was related with its anti-inflammatory activity (Tian et al., 2015).

### Anticataract Activity

*D. huoshanense* is an important traditional Chinese medicine with a protective effect on the eyes and has long been used to prepare eye protection preparations, such as Shihu Yeguang pills (Editorial Board of China Pharmacopoeia Committee 2020; Horng et al., 2021). Therefore, *D. huoshanense* is of interest for the treatment of eye diseases. Luo et al. found that crude polysaccharides from *D. huoshanense* could inhibit the oxidation pathway by the down-regulation of inducible nitric oxide synthase (iNOS) gene expression and advanced glycation end product (AGEs) formations to suppress diabetic cataract, which is an important cause of blindness worldwide (Luo et al., 2008). Especially, at 200 mg.kg^−1^.day^−1^, the crude polysaccharide significantly reduced the level of nitric oxide (NO) (70.12 ± 1.2 μmol mg^−1^ protein) and the activity of iNOS (U mg^−1^ protein), and the fluorescence intensity of AGEs was remarkably inhibited, which showed that the *D. huoshanense* polysaccharide has potential for the prevention and cure of diabetic cataract. Another study showed that the *D. huoshanense* polysaccharide significantly improved the level of GSH, decreased the content of MDA, and increased the activities of glutathione peroxidase (GSH-PX), glutathione reductase (GR), glutathione S-transferase (GST), SOD, and CAT in the lens of diabetic rats, indicating that the *D. huoshanense* polysaccharide may also prevent the development of diabetic cataracts by ameliorating oxidative stress (Li et al., 2012). In order to investigate the core structure of the *D. huoshanense* polysaccharide against cataracts, the refined polysaccharide DHPD1 was enzymatically hydrolyzed with pectinase to obtain different fragments of oligosaccharides and their anti-cataract activities were evaluated with the apoptosis model of human lens epithelial cells induced by H_2_O_2_ (Zha et al., 2017). The results showed that DHPD1-24, composed of (1 → 5)-linked-Ara*f*, (1 → 3,6)-linked-Man*p*, 1-linked-Glc*p*, (1 → 4)-linked-Glc*p*, (1 → 6)-linked-Glc*p*, (1 → 4,6)-linked-Glc*p*, (1 → 6)-linked-Gal*p,* and 1-linked-Xyl*p* in a molar ratio of 1.06:1.53:2.11:2.04:0.93:0.91:0.36:1.01, was the core structure of the *D. huoshanense* polysaccharide against cataract, and it could inhibit H_2_O_2_-induced human lens epithelial cell apoptosis through suppressing the MAPKs signaling pathway, which provided the foundation for further unraveling the structure–activity relationship of the *D. huoshanense* polysaccharide against cataract.

### Antiglycation Activity

Glycation is a non-enzymatic reaction that inserts sugar chains into macromolecules such as proteins, DNA, and lipids, to form stable covalent structures. These bound products are involved in developing diabetes, metabolic syndrome, obesity, hypertension, atherosclerosis, and Alzheimer’s disease to result in body damage. Studies have shown that *D. huoshanense* polysaccharides have significant anti-glycation effects. For example, a polysaccharide, DHP-W2, isolated from *D. huoshanense* achieved 23% inhibition of protein glycation after 3 weeks of reaction at a concentration of 0.5 mg/ml, which was similar to that of vitamin C at 0.3 mg/ml (Inhibition of protein glycosylation, 28%) (Pan et al., 2013). Luo et al. isolated DHPD1 from *D. huoshanense* and prepared DHPD1 derivatives under enzymatic degradation conditions to investigate the inhibitory effects of molecular weight alteration of the *D. huoshanense* polysaccharide on protein glycation (Zha et al., 2013). The results indicated that the anti-glycation activity of the *D. huoshanense* polysaccharide reduced with the decrease of the molecular weight.

Furthermore, the sulfated DHPD1 with a degree of substitution of 1.473 was studied, displaying that the inhibition activity of sulfated DHPD1 on protein glycation at 1.0 mg/ml was 58.5%, which was 16.2% and 52.5% higher than the same dose of aminoguanidine and DHPD1, respectively (Qian et al., 2014). Luo et al. also extracted DHPD2 from *D. huoshanense* by fractionation on the DEAE-Cellulose column and dialysis (molecular weight cut off: 8,000 Da), and simultaneously prepared sulfated DHPD2 derivatives by chlorosulfonic acid-pyridine (CSA-Pyr) method (Li et al., 2014). The results of the anti-glycation assay showed that the activity of DHPD2 was enhanced after sulfation and was more favorable at C-2 and C-6 sulfations of the glycosyl residues.

### Others

In addition to the bioactivities described previously, it was found that *D. huoshanense* also had important effects on anti-aging (Gu F. L. et al., 2021), anti-rheumatoid arthritis (Shang et al., 2021), anti-atherosclerosis (Fan et al., 2020), anti-inflammation (Ge et al., 2018; Gu FL. et al., 2021), hypoglycemic activity (Pan et al., 2014; Wang et al., 2019), regulation of intestinal flora (Xie et al., 2019a), and constipation caused by the spleen’s yin deficiency (Gan et al., 2019). However, its specific mechanism of action is still to be further explored. All in all, *D. huoshanense* is worthy for an in-depth study.

## Conclusion and Future Prospects

As the best of *Dendrobium*, *D. huoshanense* has attracted increasing attention. Currently, the materia medica research and resource aspects of *D. huoshanense* have been evident. Furthermore, the *D. huoshanense* industry is vast, with an 8.0 million m^2^ promoted planting area and 350 tons of the annual production (including flower, fresh and dry materials of *D. huoshanense*) in Lu’an of the Anhui province. Therefore, this review systematically summarized the recent research on the chemical composition and pharmacological effects of *D. huoshanense* to provide references for further research on *D. huoshanense*, as well as help in a more in-depth understanding, development, and utilization of *D. huoshanense*. Importantly, this review reveals that many aspects of *D. huoshanense* warrant further investigation. The detailed discussion is as follows.

(i) Although efficient methods for the conservation resources of *D. huoshanense* have been obtained, further studies aiming at improving bioactive secondary metabolites in cultivated *D. huoshanense* should be actively performed, which are relevant to its pharmacological activity and beneficial for its commercialization.
(ii) *Via* different isolation methods, *D. huoshanense* polysaccharides with different structures can be obtained so that *D. huoshanense* polysaccharides are worthy of continued research.
(iii) Compared with other natural polysaccharides, the pharmacological effects of *D. huoshanense* polysaccharides are still under-researched, which need to be comprehensively studied to improve the medicinal value of *D. huoshanense.*
(iv) There are many studies on immunomodulation of the *D. huoshanense* polysaccharide, which shows desirable results, but its druggability has not been studied in depth.
(v) Compared with *D. officinale*, there are fewer studies on the small molecule chemical compositions of *D. huoshanense*, and there remains significant room for further research.
(vi) Many studies on the pharmacological activity and chemical compositions of *D. huoshanense* have not clearly marked the origin, including the cultivation mode and growth years, which is unfavorable to the systematic study of *D. huoshanense* and should be paid more attention.

These findings provide guidance for further research on *D. huoshanense* and encourage researchers to develop new functions and utilization.

## Glossary

AGEs: advanced glycation end products
AGEs: advanced glycation end products
ALT: alanine aminotransferase
AST: aspartate aminotransferase
CAT: catalase
CSA-Pyr method: chlorosulfonic acid-pyridine method
D. chrysotoxum: Dendrobium chrysotoxum
D. huoshanense: Dendrobium huoshanense
D. moniliforme: Dendrobium moniliforme
D. nobile: Dendrobium nobile
D. officinale: Dendrobium officinale
GC-MS: gas chromatography-mass spectrometry
GSH: L-glutathione
GSH-PX: glutathione peroxidase
GST: glutathione S-transferase
GR: glutathione reductase
HMBC: heteronuclear multiple bond correlation
H_2_O_2_: Hydrogen peroxide
HPLC-GPC: high performance gel permeation chromatography
HSQC: heteronuclear singular quantum correlation
IFN-γ: interferon-γ
IL-1β: interleukin-1β
IL-12 p40: interleukin 12 p40
IL-10: interleukin 10
IL-6: interleukin 6
iNOS: inducible nitric oxide synthase
IR: infrared radiation
LDH: lactate dehydrogenase
MAPKs: mitogen-activated protein kinases
MCP-1: monocyte chemoattractant protein-1
MDA: malonaldehyde
MIP-2: macrophage inflammatory protein-2
NF-κB: nuclear factor-kappa B
NK cells: natural killer cells
NKT cells: natural killer T cells
NMR: nuclear magnetic resonance
NO: nitric oxide
8-OhdG: 8-hydroxy-20-deoxyguanosine
PI3 K/Akt: phosphoinositide 3-kinase-akt
p-IκBα: phosphorylated IκBα
SOD: superoxidedismutase
TGF-β1: transforming growth factor β1
TNF-α: tumor necrosis factor-α
T-AOC: total antioxidant capacity
